# Transcriptional Pathways Predisposing to Cancer Oxidative Stress Sensitivity and Resistance Are Shared Between Hydrogen Peroxide and Cold Gas Plasma but Not Hypochlorous Acid

**DOI:** 10.3390/cancers17020319

**Published:** 2025-01-20

**Authors:** Debora Singer, Sander Bekeschus

**Affiliations:** 1 Department of Dermatology and Venerology, Rostock University Medical Center, Strempelstr. 13, 18057 Rostock, Germany; 2 ZIK *plasmatis*, Leibniz Institute for Plasma Science and Technology (INP), Felix-Hausdorff-Str. 2, 17489 Greifswald, Germany

**Keywords:** CAP, kINPen, low-temperature plasma, medical gas plasma technology, non-thermal plasma, oxidative stress, reactive oxygen species

## Abstract

Oxidative stress is universal to all cell types, including cancer. It is elicited by a surplus of reactive oxygen species (ROS) or a reduced cellular ability to defend against those. At low levels (oxidative eustress), this induces altered cellular signaling, while at higher levels (oxidative distress), cellular toxicity and non-specific redox signaling become apparent. While oxidation-induced cell death is a hallmark of many cancer therapies, including ROS-producing radiotherapy, some chemotherapies and targeted therapies, photodynamic therapy, and recently emerging physical modalities such as medical gas plasma (a multi-ROS generating technology), less is known about the transcriptional profiles predisposing cancer cells to oxidative demise. In particular, which genes are associated with resistance or sensitivity to ROS overload and subsequent toxicity has not been systematically investigated. Moreover, it is unclear if there are differences between oxidant types, such as hydrogen peroxide and hypochlorous acid. To this end, we here employed 35 cell lines of various origins (e.g., adenocarcinoma, melanoma, leukemia, squamous cell carcinoma, and neuroblastoma). We first performed in-house transcriptomic analysis to assess baseline transcriptional profiles. Second, all cell lines were exposed to four different ROS concentrations of either hydrogen peroxide, hypochlorous, or gas plasma exposure. Third, correlation analysis was performed to identify genes associated with (i) oxidative stress sensitivity, (ii) oxidative stress resistance, and (iii) similarities and/or differences between the different oxidative stress inducers. Intriguingly, distinct gene sets were found for all treatments, and there was a striking difference between hydrogen peroxide and hypochlorous acid, suggesting different modes of action of both oxidants.

## Simple Summary

Reactive oxygen species (ROS) are more or less present inside and in the environment of all cell types. ROS can cause positive (e.g., signaling) or negative (e.g., toxic) effects depending on their concentration. Cytotoxic effects of such molecules are harnessed in many anticancer therapies. Nevertheless, different cancer cell types react to the ROS to varying extents. Until now, little has been known about the expression of genes associated with lower or higher toxicity in the different cell types. In the present study, gene expression data of 35 cell lines were obtained, and the sensitivity of these lines toward three ROS types was tested, aiming to find associated genes and draw comparisons among the different ROS. The most significant difference was found between hydrogen peroxide and hypochlorous acid sensitivity-associated genes, implicating different toxicity mechanisms of these two ROS.

## 1. Introduction

Reactive oxygen species (ROS) are essential mediators in physiological and pathological processes [1]. Most organisms generate ROS via, e.g., NADPH-oxidase (NOX), which produces superoxide, and nitric oxide synthases (NOS), producing nitric oxide. ROS are involved in cellular signaling processes at physiological concentrations, such as development, aging, or immunity (oxidative eustress). In contrast, at higher concentrations, irreversible damage of important biomolecules can lead to cellular dysfunction or cell death (oxidative distress) [2]. At constantly higher ROS levels, the cellular distress conditions necessary for chronic inflammation and cancer can be induced [3]. The long-lived non-radical ROS H_2_O_2_ is a critical regulator of cellular processes, where it is mainly generated via the spontaneous or superoxide dismutase (SOD)-mediated conversion of superoxide. H_2_O_2_ is known to contribute to oncogenesis and tumor progression [4] in chronically inflamed environments. From H_2_O_2_, another potent ROS—hypochlorous acid (HOCl)—can be produced through a myeloperoxidase (MPO-) controlled reaction with chloride anions [5]. This occurs during antimicrobial defense, chronic inflammation, and tumor microenvironments (TME) rich in tumor-infiltrating neutrophils [6,7]. At the same time, ROS-induced cytotoxic effects can be harnessed for therapeutic purposes, e.g., in cancer therapy [8]. This is seen in radiotherapy [9], photodynamic therapy [10], and multi-ROS-generating medical gas plasma technology [11], which all have been noted to deliver ROS at tumor-toxic concentrations. Medical gas plasma is a partially ionized gas produced at about body temperature and is currently used for medical purposes such as wound healing supportive therapy [12].

Although oxidative stress is a major hallmark of cancer and many therapies, there is relatively little known about factors predisposing tumor cells to oxidation-induced cell death. A large body of evidence has investigated the responses of tumor cells to oxidative stress induced via various sources, including chemicals and physical treatment modalities, unraveling multiple signaling pathways as a consequence of oxidative stress [13,14]. In addition, there is still the traditional view pertaining to the idea that oxidative stress intensity dominates over the type of ROS or oxidant, i.e., that there are similar mechanisms of action involved if, e.g., 50% cancer cell killing is achieved in cell culture, independent on the type of oxidant. However, no studies are available that address the question of a priori ROS sensitivity or resistance, i.e., how the basal cellular transcription associates with the (non-) fitness of tumor cells to cope with oxidative stress. To this end, we here tested 35 cell lines in vitro by assessing the whole-genome transcriptomic profile generated in-house, associating this with oxidative stress sensitivity and resistance, and investigating similarities and differences between three oxidative stress inducers. For the first time, we report striking differences between hydrogen peroxide and hypochlorous acid-induced cancer cell demise.

## 2. Materials and Methods

### 2.1. Cell Culture

In this study, 35 human cell lines from different origins were cultured, including malignant melanoma (SK-MEL-19, SK-MEL-29, SK-MEL-63, SK-MEL-147, Ma-Mel-86a, MeWo, 501-Mel, and A375), leukemia (THP-1, TK6, HL-60, U937 and Jurkat), adenocarcinoma (Capan-1, HeLa (wild type and derivative 2c), HT-29, MCF7, MDA-MB231, OVCAR-3, A549, MIA-PaCa-2, PaTu-T, PaTu-S, PC3, SK-OV-3, SW480, TOV-112D, and TOV-21G), squamous cell carcinoma (A431, SCC-13, SCL-1, and SCL-2), neuroblatoma (SH-SY5Y), and non-malignant keratinocyte (HaCaT) cells. The cell lines were purchased from ATCC, or HeLa cells (wt and 2c) were kindly provided by Christopher Lillig (Greifswald University Medical Center, Greifswald, Germany). All cell lines were cultured in an S2 laboratory under standard conditions (37 °C, 5% CO_2_, and 95% humidity) using their respective culture media.

### 2.2. Metabolic Activity

In a 96-well plate (Sarstedt, Sarstedt, Germany), 1 × 10^4^ cells were seeded in 100 μL fully supplemented cell culture media (Roswell Park Memorial Medium 1640 supplemented with 10% fetal bovine serum (FBS), 1% L-glutamine, and 1% penicillin and streptomycin) per well and incubated overnight. The following day, the culture medium was replaced with fresh cell culture medium. Subsequently, the cells were exposed to the gas plasma of an atmospheric pressure argon plasma jet (kINPen; neoplas, Greifswald, Germany) operated at two standard liters per minute (for either 30 s, 60 s, or 120 s) or different concentrations of the two oxidants H_2_O_2_ (1 μM, 10 μM, 100 μM, or 1000 μM) or HOCl (0.1 mM, 1 mM, 10 mM, or 100 mM), or were left untreated. Twenty hours after treatment, a final concentration of 100 μM resazurin was added to each well, and the plates were incubated for 4 h at 37 °C. To quantify the metabolic activity of the cells, the turnover of resazurin to fluorescent resorufin was measured using an Infinite F200 microplate reader (Tecan, Männedorf, Switzerland) at λ_ex_ 560 nm and λ_em_ 590 nm. Mean fluorescent intensities of treated cells were normalized to untreated cells and log-transformed to calculate IC_25_ concentrations using nonlinear regression.

### 2.3. Whole-Genome Transcriptomic Analysis

Transcriptomic data obtained for a previous study [15] were re-analyzed to correlate gene expression with the cell lines’ sensitivity to medical gas plasma, H_2_O_2_, and HOCl. Briefly, cells were grown in cell culture flasks and harvested using accutase for RNA analysis. At least 5 × 10^5^ cells were washed with PBS, and RNA was isolated using the RNA Mini kit (Bio&Sell, Feucht, Germany). Isolated RNA concentrations were quantified using the spectrophotometer Nanodrop 2000C (Thermo Fisher Scientific, Bremen, Germany) and stored at −80 °C until further preparation. A single-color, chip-based whole-transcriptome microarray covering 55,077 distinct biological features (SurePrint G3 Human CGH Microarray 8 × 60 K; Agilent Technologies, Waldbronn, Germany) was used for genome-wide profiling. The spike-in mix was added to 100 ng RNA and transcribed into cDNA, which was amplified using the Low Input Quick Amp Labeling One Color component. The cDNA was then transcribed to cRNA labeled with the fluorescent dye cyanine 3 (Cy3). Cy3-cRNA was purified and hybridized onto the microarray chip for 17 h at 65 °C. After washing, the chips were scanned using an Agilent SureScan device, and scans were evaluated using the Agilent Feature Extraction Software. At least three independent biological replicates were sampled per cell line and averaged to 35 transcriptomes when analyzed using the GeneSpring software (version 14.9.1; Agilent Technologies, Waldbronn, Germany).

### 2.4. Bioinformatic Analysis

The GeneSpring software was used to perform robust multi-array (RMA) normalization followed by normalization on ribosomal protein L13a (*RPL13A*) housekeeping gene expression [16,17]. The normalized expression of 26,610 gene expression targets was Spearman-correlated against the IC_25_ values for each cell line’s medical gas plasma, H_2_O_2_, or HOCl exposure using the TipCo Spotfire software version 7.8 (PerkinElmer, Hamburg, Germany). Only genes showing significant correlation (*p* < 0.05) and whose IDs could be mapped using the open-access PANTHER (Protein ANalysis Through Evolutionary Relationships) database were analyzed subsequently. The PANTHER database was further used to perform a functional classification of the top correlating genes based on Gene Ontology (GO) terms. The STRING database was used to identify shared networks of correlating genes. Functional enrichment was visualized using the open-access Reactome analysis tool.

## 3. Results

### 3.1. Gas Plasma and H_2_O_2_ Showed High Correlation While HOCl Cytotoxicity Differed Strongly

**Figure 1:**
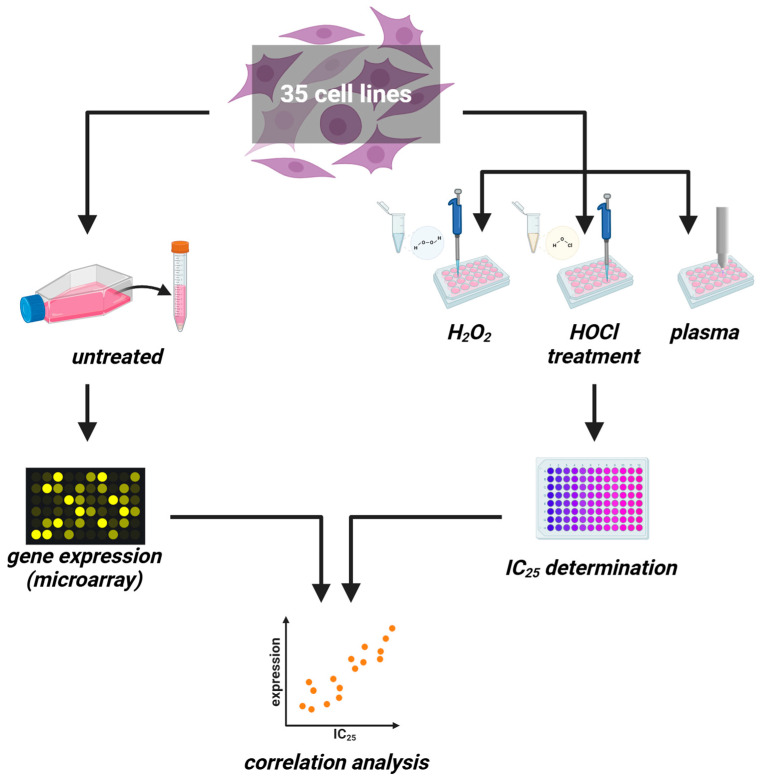
**Study protocol.** (**left**) Thirty-five untreated cancer cell lines were cultured to harvest RNA for transcriptomic microarray analysis. (**right**) In parallel, the same cell lines were treated with increasing H_2_O_2_ or HOCl concentrations or increasing medical gas plasma treatment times for IC_25_ determination. Both datasets were correlated subsequently.

**Figure 2:**
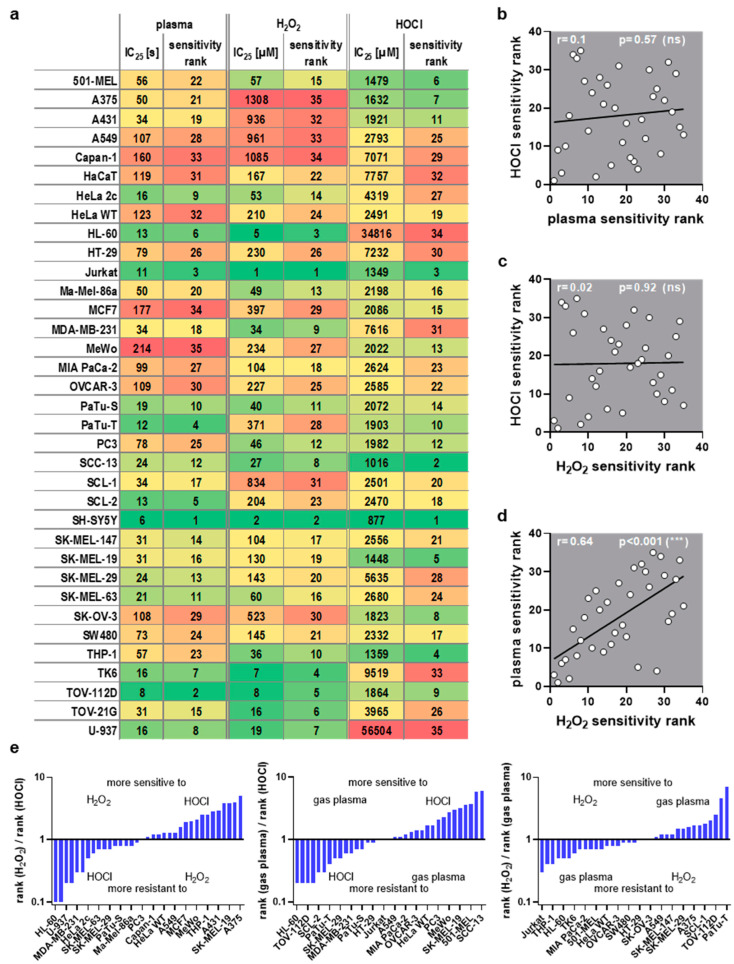
**ROS sensitivity comparison.** (**a**) IC_25_ values and assigned sensitivity ranks for medical gas plasma, H_2_O_2_, and HOCl, for cell lines being sorted alphabetically (each column has been color-coded for values only within each column from low values being green and high values being red); (**b**–**d**) Spearman correlation of (**b**) HOCl vs. plasma sensitivity, (**c**) HOCl vs. H_2_O_2_ sensitivity, and (**d**) plasma vs. H_2_O_2_ sensitivity showed a strong correlation between plasma and H_2_O_2_. (**e**) rank ratios of the three ROS treatments sorted from low to high for the three comparisons (for clarity, only every second cell line is labeled; missing entities can be retrieved from (**a**)).

**Figure A1:**
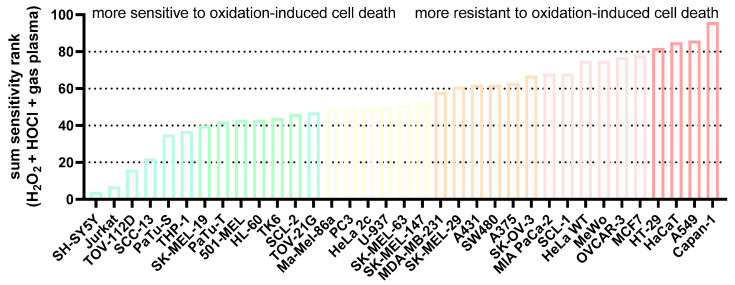
**Sum sensitivity scores.** The sensitivity scores of the IC_25_ ranks of all cell lines for each ROS treatment (H_2_O_2_, HOCl, gas plasma) were added to display the overall rather sensitive and relatively resistant cells to oxidation-induced cell death.

This study investigated transcriptomic profiles of 34 human cancer cell lines and HaCaT keratinocytes to identify basal gene expression patterns associated with sensitivity or resistance to different ROS treatments. To this end, inhibitory concentrations (IC_25_) of medical gas plasma exposure (three treatment times), H_2_O_2_ (four concentrations), and HOCl treatments (four concentrations) were determined 24 h post-exposure in all 35 cell lines (Figure 1, left). In parallel, the basal gene expression of all the same cell lines was quantified in an in-house transcriptomic microarray study (Figure 1, right) to correlate the resulting datasets with the three IC_25_ datasets for each of the 35 cell lines tested and against each other. Before correlation analysis was performed, IC_25_ values were compared across the tested cell lines and used to assign sensitivity ranks. A wide range between the most oxidation-induced cell death-sensitive cell line and the most oxidation-induced cell death-resistant cell lines was found in all three treatment regimes tested. This was the highest for H_2_O_2_ sensitivity (>1000-fold). At the same time, for gas plasma exposure, there was a factor of 35 between the gas plasma exposure time necessary to induce the IC_25_ in the most sensitive compared to the most resistant cell line, respectively (Figure 2a). For hypochlorous acid exposure, this factor was 64. The high concentrations pertain to the FBS being present in the cell culture media during exposure to hypochlorous acid, scavenging a larger share of the oxidant and requiring higher concentrations of HOCl to achieve cytotoxic effects. The next question was whether the three oxidative stress inducers shared similarities in terms of sensitivity ranks concerning the 35 cell lines investigated. Interestingly, no significant correlation was detected between HOCl and gas plasma exposure (Figure 2b) (r = 0.10), pointing to different mechanisms of action of both treatments. Along similar lines, no significant correlation was identified between HOCl and H_2_O_2_ exposure (Figure 2c) (r = 0.02), i.e., cell lines sensitive to HOCl are not necessarily sensitive to H_2_O_2,_ and vice versa, indicating that both oxidants are effective via different signaling pathways. Strikingly, there was a high (r = 0.64) and significant (*p* < 0.001) correlation between the cell lines’ sensitivity to H_2_O_2_ and gas plasma exposure (Figure 2d), suggesting that H_2_O_2_ is a primary oxidant responsible for the action of gas plasma treatment in cancer cells. Moreover, to illustrate the divergent and congruent behavior of each cell line towards the three oxidant treatments, ratios of the ranks (Figure 2a) were calculated and labeled for any other cell line (Figure 2e). In addition, the sum of all ranks was calculated for each of the cell lines tested to indicate the general nature of the cell line being relatively sensitive or relatively resistant to oxidative stress-induced cellular demise (Figure A1).

### 3.2. Gas Plasma and H_2_O_2_ but Not HOCl Showed Overlapping Genes Associated with Oxidative Stress Sensitivity or Resistance

**Figure 3:**
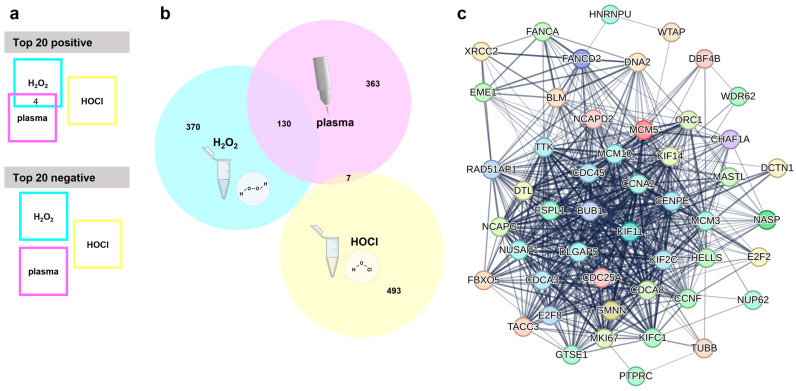
**Transcriptomic analysis.** (**a**) Visualization of overlapping genes in the top 20 genes with positive or negative correlation with medical gas plasma, H_2_O_2,_ or HOCl sensitivity shows four shared genes in the top 20 positive and no common genes in the top 20 negatively correlating genes; (**b**) Venn diagram used to check for shared genes in the top 500 overall (positively and negatively) correlating genes from medical gas plasma; H_2_O_2_ or HOCl sensitivity correlations show wide overlap between medical gas plasma and H_2_O_2_ sensitivity; (**c**) STRING network of 39 cell cycle-related genes out of 130 shared genes from plasma and H_2_O_2_ overlap.

The next question was which genes are associated with an inherent cancer cell sensitivity or resistance to oxidant-induced cell death. Baseline expression data, i.e., from untreated cell lines were generated using whole-gene transcriptomics arrays. The relative expression of each gene across all cell lines was then correlated with the corresponding IC_25_ values of one of the ROS treatments. This was then repeated for the other two ROS treatments. After removing uncharacterized genes and those without functional annotations using bioinformatic analysis, a total of 6227 genes were found to correlate significantly (*p* < 0.05) either positively or negatively to the gas plasma IC_25_ values. Lower numbers of positive or negative significantly correlating genes were found for the other two ROS treatments, namely H_2_O_2_ (2477 genes in total) and HOCl (997 genes in total), against the respective IC_25_ values. The top 20 genes with positive correlation, as well as the top 20 genes with negative correlation (sorted according to their *p*-values, starting with those having the lowest *p*-value), were listed for each of the treatments, i.e., gas plasma (Table 1), H_2_O_2_ (Table 2), and HOCl (Table 3). These top 20 correlating genes were then compared across the three ROS treatments (Figure 3a). Interestingly, there was a modest overlap only between the positively correlating genes of gas plasma and H_2_O_2_. Those genes were *ME1*, *BLVRB*, *AREG,* and *CFAP300*. For H_2_O_2_ and HOCl, gas plasma and HOCl, and H_2_O_2_ and gas plasma and HOCl, no overlapping genes were identified among the top 20 positively correlating genes. Likewise, no overlap in the top 20 lists of negatively correlating genes was found between any of the three ROS treatments. A more global perspective that included the top 500 significantly regulated genes (both positive and negative correlation) revealed a similar result (Figure 3b). Gas plasma and H_2_O_2_ showed a good overlap (130/500 genes) of about 20% of the significantly correlating genes associated with oxidative stress sensitivity or resistance. In comparison, the overlap between gas plasma and HOCl was minimal (7/500 genes) or even absent (0/500 genes) in the case of H_2_O_2_ and HOCl. The 130 shared gas plasma and H_2_O_2_ genes were further analyzed using the STRING database. This revealed an extensive network of 39 of 130 (30%) genes involved in the cell cycle (Figure 3c), pointing to a highly relevant role of cell cycle-related genes potentially being involved in the sensitivity or resistance to gas plasma and H_2_O_2_-mediated toxicity in vitro. No such network was found for the seven shared genes of gas plasma and HOCl sensitivity.

### 3.3. Gene Ontology Classification

**Figure 4:**
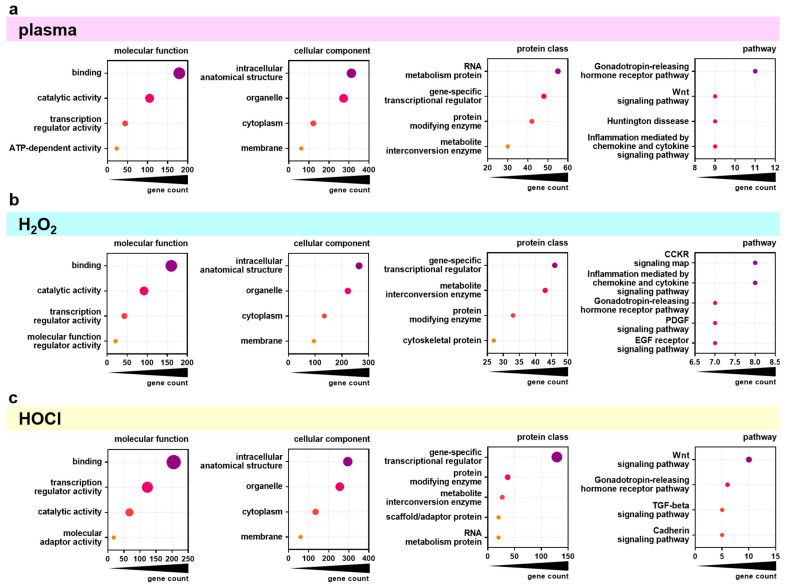
**PANTHER analysis of differentially regulated genes.** (**a**–**c**) Major groups in molecular function, cellular component, protein class, and pathway as found by classification analysis using gene ontology (PANTHER) for the overall top 500 genes significantly correlating with gas plasma (**a**), H_2_O_2_ (**b**), and HOCl (**c**) sensitivity.

**Figure A2:**
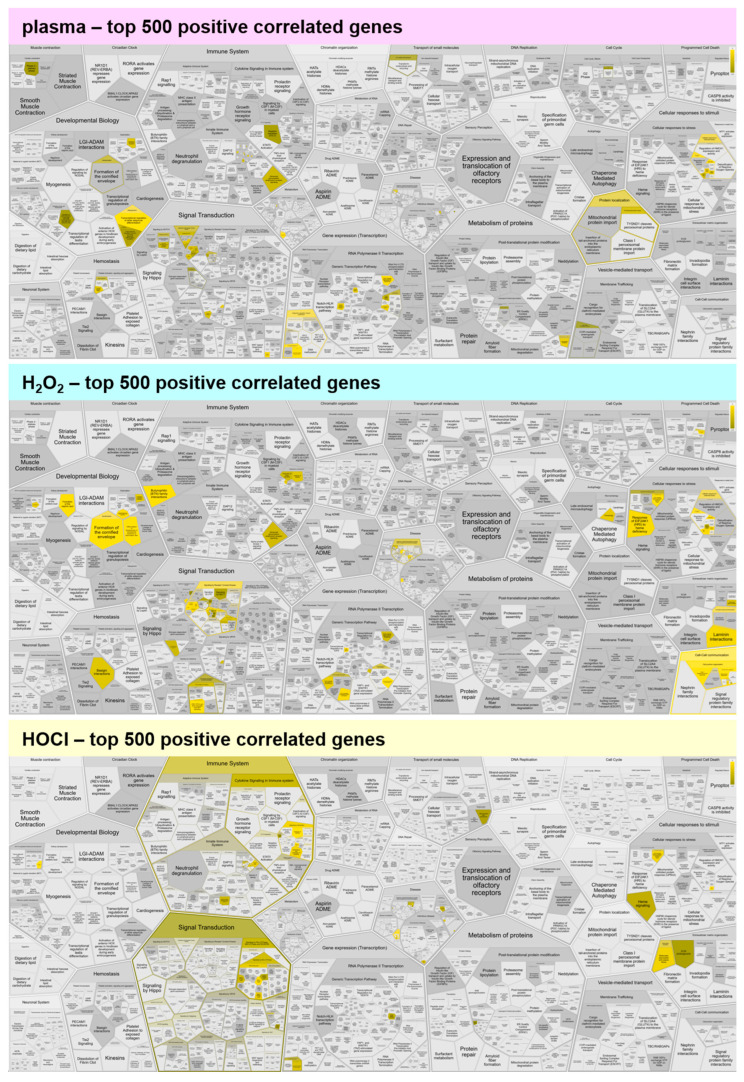
**Functional enrichment analysis of positively correlating genes.** Reactome analysis of the top 500 positively correlating genes showed few functional enrichment spots for medical gas plasma, H_2_O_2_, and HOCl. Yellow colors indicate categories in which correlated genes fall, and the dark to light color gradient indicates the number of such genes from few to many.

**Figure A3:**
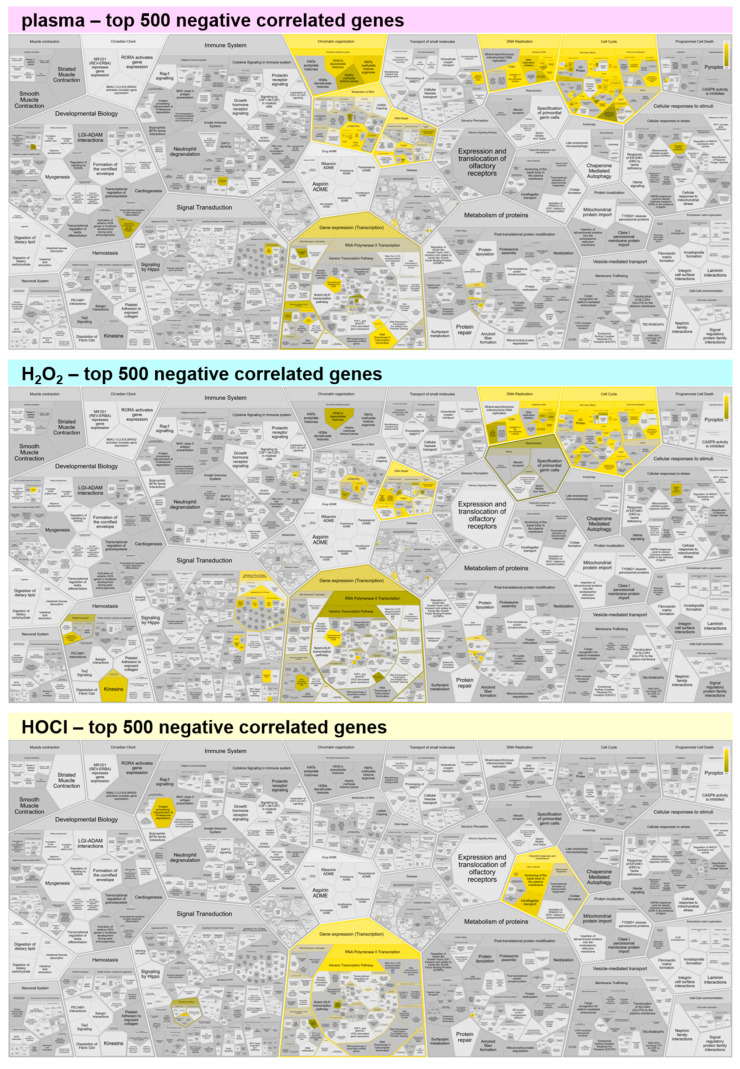
**Functional enrichment analysis of negatively correlating genes.** Reactome analysis of the top 500 negatively correlating genes showed comparable functional enrichment spots for medical gas plasma and H_2_O_2_, while HOCl shows only little enrichment. Yellow colors indicate categories in which correlated genes fall, and the dark to light color gradient indicates the number of such genes from few to many.

**Figure A4:**
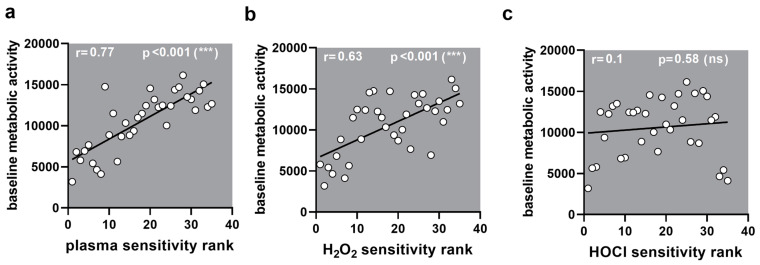
**Correlation to metabolic activity.** (**a**–**c**) Correlation of sensitivity ranks with baseline metabolic activity showed a significant correlation for (**a**) medical gas plasma (0.77) and (**b**) H_2_O_2_ (0.63) but not (**c**) HOCl (0.10).

Next, the top 500 overall correlating genes were analyzed using the PANTHER database for functional classification based on gene ontology terms. The top four categories (or five in case of several categories with equal gene counts) of the topics categories of molecular function, cellular component, protein class, and pathway were identified for the significantly positively or negatively correlating genes for gas plasma (Figure 4a), H_2_O_2_ (Figure 4b), and HOCl (Figure 4c) exposures and IC_25_ values. The molecular functions of the significantly correlating genes were mainly *binding*, *catalytic activity*, and *transcription regulator activity* for all three ROS treatments. The classification of the categories of the cellular components was similar not only in content (*intracellular anatomical structure*, *organelle*, *cytoplasm*, *membrane*) but also in rank across all three ROS exposure groups. For the category of protein classes, the top categories were mainly the same in all three ROS exposure groups but to different extents. Gas plasma-correlating genes showed the highest gene count in the *RNA metabolism,* while H_2_O_2_- and HOCl-correlating genes contained more *gene-specific transcriptional regulators*. Nevertheless, the gene count in *gene-specific transcriptional regulators* was three times higher in HOCl- than in H_2_O_2_- correlating genes. The most significant differences were visible in the classification of associated signaling pathways. Here, gas plasma-correlating genes were primarily associated with the *Gonadotropin-releasing hormone receptor pathway*. In contrast, H_2_O_2_ was mainly related to the CCKR signaling map and *inflammation mediated by chemokine and cytokine signaling* pathways. Signaling with HOCl was related dominantly to the Wnt signaling pathway, which was also a high category in the gas plasma group. Functional enrichment separated to positively and negatively correlating genes was further visualized using the Reactome analysis tool. Only a few categories across various topics with significantly enriched gene function were identified as overlapping for positively correlating genes comparing the three ROS treatment groups (Figure A2). By contrast, the negatively correlating genes showed more functional enrichment, especially comparable *DNA Replication* and *Cell Cycle* patterns for gas plasma and H_2_O_2_ (Figure A3). Genes correlating negatively to HOCl showed no significant enrichment in these categories except for *Gene expression (Transcription)*. Based on the results of functional classification and enrichments, the association of general metabolic activity of the cell lines with ROS sensitivity was tested by the Spearman correlation of baseline metabolic activity obtained from resazurin assays with sensitivity ranks (Figure A4). A strong and significant correlation was found between gas plasma sensitivity (Figure A4a) and H_2_O_2_ sensitivity (Figure A4b), while no correlation between metabolism and HOCl sensitivity could be detected.

## 4. Discussion

Little is known about predetermining factors such as baseline gene expression associated with either the enhancement or the decline of oxidative stress sensitivity in cancer cells. To this end, the basal gene expression of 35 cell lines from different origins (adenocarcinoma, melanoma, leukemia, squamous cell carcinoma, neuroblastoma, and keratinocyte) was analyzed using in-house whole-transcriptome microarrays. Gene expression was correlated with resistance and sensitivity against medical gas plasma-delivered ROS mixture and two well-known long-lived oxidants, H_2_O_2_ and HOCl. In parallel, the cells’ sensitivity was examined based on their metabolic activity towards the well-explored ROS hydrogen peroxide (H_2_O_2_) [18], the inflammation-associated hypochlorous acid (HOCl) [19], and the therapeutically applicable medical gas plasma (argon plasma jet kINPen) producing a multitude of ROS [20]. Determined inhibitory concentrations (IC_25_) were correlated against relative gene expression, and significantly correlating genes were compared among the three tested oxidative stress inducers. Interestingly, when cell line sensitivity ranks were compared, a significant correlation was found between H_2_O_2_ and medical gas plasma sensitivity, while HOCl did not correlate with the other two ROS treatments. Comparing the top 500 significantly correlating genes, 130 genes matched between H_2_O_2_ and medical gas plasma, while for HOCl, only seven matched with medical gas plasma, and none with H_2_O_2_.

The ROS-mediated cytotoxic effect of medical gas plasma at sufficient treatment times against cancer cells was well described during the last decade. Nevertheless, different cell types show varying responses, ranging from high sensitivity to near resistance. This study aimed to increase the understanding of conditions that cause predestined cells to be more or less vulnerable to gas plasma-induced cytotoxicity. Thirty-five human cell lines were screened for their sensitivity towards medical gas plasma (kINPen)-produced ROS-mixture and H_2_O_2_ and HOCl alone. Basal transcriptome data of the cell lines were correlated with the inhibitory concentrations to find gene expression patterns associated with gas plasma sensitivity and compared to correlation with the other two long-lived oxidants, H_2_O_2_ and HOCl.

IC_25_ values, used to quantify the cells’ sensitivity to the tested ROS, varied considerably among the cell lines. The sensitivity ranking used to compare the different ROS showed that the cell lines reacted entirely differently depending on what kind of ROS was applied, underlining that oxidative stress can be complex and that not all ROS can be lumped together. Variations in cells’ capability to tolerate toxicity induced by different oxidants were also observed on a smaller scale in a previous study using three different abdominal cancer cell lines and HaCaT cells [21]. In the present study, HOCl was the most different from H_2_O_2_ and gas plasma, while H_2_O_2_ and gas plasma sensitivities showed a good correlation as previous studies suggested [22,23] and hence were similar but not the same.

The highest amount of significantly correlating genes was found for the gas plasma-induced toxicity, followed by H_2_O_2_ and the least HOCl sensitivity-correlating genes. In total, more genes with negative correlation were found in all three ROS groups, meaning that more genes showed relatively high expression in the sensitive cell lines and lower expression in the resistant cell lines. Four genes were shared within the top 20 positively correlating gas plasma and H_2_O_2_ sensitivity genes. *ME1, BLVRB, CFAP300,* and *AREG* were higher expressed in gas plasma and H_2_O_2_-resistant cell lines. The NADP-dependent malic enzyme (ME1) plays a role in intermediary metabolism by generating NADPH for fatty acid synthesis [24]. By controlling the cellular levels of NADPH, it is involved in redox homeostasis, and a role of ME1 in tumor progression through its ability to reduce intracellular ROS levels is described [25,26,27]. Biliverdin reductase B (BLVRB) is an NADPH-dependent enzyme that is also involved in redox homeostasis by the reduction of biliverdin to bilirubin within heme degradation but can also reduce several other substrates [28]. Amphiregulin (AREG) is part of the epidermal growth factor (EGF) family, and it has a dual role due to the possibility of inducing proliferation or inhibiting growth [29]. The overexpression of AREG was found in numerous cancers where it was associated with tumor survival and chemotherapy resistance [30].

Looking further at the top 500 correlating genes of all three ROS groups, it was impressive that medical gas plasma and H_2_O_2_ had more than one-quarter in common. These 130 overlapping genes revealed a network of 39 genes involved in cell cycle processes. Within this network, we found the well-known marker of proliferation ki-67 (MKI67), often used for quantifying the growth rate of tumor cells [31]. Another interesting target showing higher basal expression in gas plasma and H_2_O_2_-sensitive cell lines was cyclin A2 (CCNA2), which regulates the cell cycle by binding cyclin-dependent kinases and is regarded as a prognostic marker of different cancer types [32,33]. Further components of the minichromosome maintenance complex (MCM3, MCM5, MCM10) which form the helicase essential in the initiation of the DNA replication, cell division cycle (CDC25A, CDC45), and associated (CDCA3, CDCA8) proteins involved in replication and cell cycle progression, as well as kinesin family members (KIF2C, KIF14, KIFC1) promoting chromosome segregation and cytokinesis, were associated with enhanced sensitivity to gas plasma and H_2_O_2_.

The enrichment of DNA replication and cell cycle within the negatively correlating genes of gas plasma and H_2_O_2_ sensitivity was underlined by the results of the re-evaluation of metabolic activity assays. Generally, a high basal metabolic activity can be regarded as a marker of fast cell proliferation, leading to an increased turnover of resazurin [34]. The correlation of the baseline metabolic activity from these assays with sensitivity ranks validated that gas plasma and H_2_O_2_ sensitivity are associated with replication and cell cycle progression as previously suggested [35,36,37,38]. In contrast, HOCl sensitivity could not be attributed to this, and the mechanisms of action governing HOCl-mediated toxicity remain to be explored in future studies.

**Figure 5:**
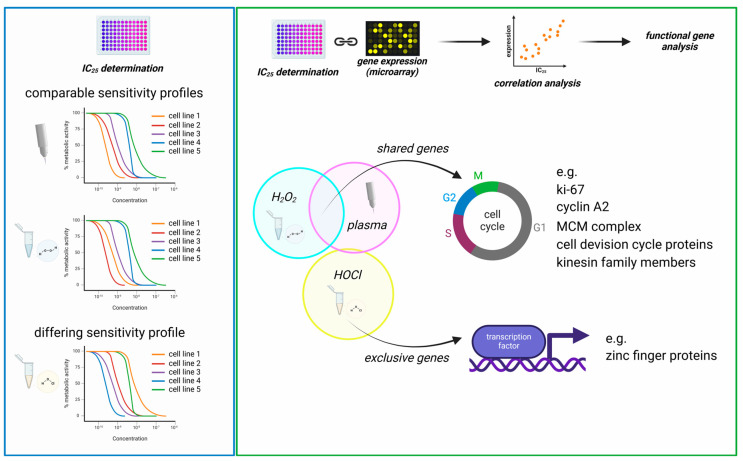
**Graphical summary.** (**left**) The comparison of sensitivity patterns (correlation of sensitivity ranks) of the 35 tested cell lines showed comparable cytotoxicity mediated by medical gas plasma and H_2_O_2_ (strong correlation), while HOCl cytotoxicity differed markedly (no correlation); (**right**) the linkage of IC values with transcriptomic data via Spearman correlation revealed distinct gene sets for all treatments but showing considerable overlap of H_2_O_2_- and gas plasma-correlating genes. Particularly, cell division-related genes were identified by functional analysis to correlate strongly with H_2_O_2_ and gas plasma-induced toxicity. In contrast, HOCl correlating genes differed from these profiles, including various transcriptional regulators.

The utilization of ROS to treat cancer cells has been studied extensively in recent years. Although it is known that ROS play a role in the development and progression of cancer [39], elevated levels of intracellular ROS are considered to make cancer cells also vulnerable to additional ROS stimuli. There is broad evidence that cancer cells can be killed with these species [40], e.g., by H_2_O_2_-induced apoptosis [41], and novel therapies targeting ROS are investigated for anticancer treatment [8,42]. In this field, a specific selectivity of ROS targeting cancer cells over normal cells is widely assumed. This might be mitigated by our findings, showing that significant differences in the vulnerability to ROS are present even within different cancer cells. The present study highlights the importance of understanding markers contributing to the sensitivity or resistance of cancer cells against the different types of ROS that might be targeted in such therapies (Figure 5). This will help, e.g., deciding for certain novel therapy approaches like inhibiting myeloperoxidase (MPO) [43] or optimizing tunable applications like the medical gas plasma technology [44].

## 5. Conclusions

With this study, we aimed to identify basal gene expression patterns to investigate a priori the enhanced or reduced sensitivity of 34 cancer cell lines to an oxidation-induced cell death mediated via H_2_O_2_, HOCl, or medical gas plasma technology exposure. We showed that an increased expression of cell cycle progression-related genes was associated with a higher vulnerability for gas plasma-induced cytotoxicity. Additionally, we found that the cells’ sensitivity to gas plasma correlates with H_2_O_2_ sensitivity and is associated with similar gene expression profiles, presumably predisposing them to oxidation-induced cell death. In contrast, HOCl sensitivity differed markedly from H_2_O_2_ (and gas plasma) effects and could not be related to a priori alterations in cell cycle progression processes. The identified gene transcription targets might serve as a valuable basis for further investigation to shed more light on the underlying mechanisms of oxidative stress resistance in tumor cells and beyond.

## Figures and Tables

**Table 1:** **Medical gas plasma—top 20 significantly correlating genes.** A total of 6227 genes were found to correlate significantly (*p* < 0.05) to gas plasma-induced toxicity. Gene names, *p*-values, and Spearman r of the top 20 genes (based on *p*-value) showing positive or negative correlation with gas plasma sensitivity are displayed in this table.

Plasma Positive Correlation			Plasma Negative Correlation		
Gene	*p*-Value	r	Gene	*p*-Value	r
*ME1*	4.39E-05	0.63	PSMC2	3.63E-10	−0.84
*CSNK1A1L*	5.65E-05	0.63	GTSE1	6.72E-08	−0.77
*DCTN1*	1.32E-04	0.60	PPP2R3B	1.23E-07	−0.76
*BLVRB*	2.99E-04	0.58	NAA40	2.16E-07	−0.75
*CFAP36*	6.47E-04	0.55	CEP135	3.27E-07	−0.74
*CAPZA2*	9.08E-04	0.54	SAFB	4.52E-07	−0.74
*SOCS6*	1.01E-03	0.53	CXXC1	5.03E-07	−0.73
*KRTAP6-2*	1.07E-03	0.53	MLF1IP	6.11E-07	−0.73
*AREG*	1.26E-03	0.52	FAM193A	7.40E-07	−0.73
*ASPH*	1.51E-03	0.52	ILF3	7.51E-07	−0.73
*DEPP1*	1.53E-03	0.52	RBM15	1.06E-06	−0.72
*CFAP300*	1.56E-03	0.51	RBM10	1.09E-06	−0.72
*MOS*	1.76E-03	0.51	NOP14	1.37E-06	−0.72
*FTHL17*	1.78E-03	0.51	RBM14	1.39E-06	−0.72
*PDK4*	1.81E-03	0.51	AGAP2	1.61E-06	−0.71
*CACNG6*	1.88E-03	0.51	WDR82	1.82E-06	−0.71
*PTGR1*	1.88E-03	0.51	MCM5	2.00E-06	−0.71
*PTCH2*	1.93E-03	0.51	MYLK2	2.11E-06	−0.71
*TMEM52*	2.31E-03	0.50	SRRT	2.20E-06	−0.71
*GPR31*	2.57E-03	0.49	MBD3	2.23E-06	−0.71

**Table 2:** **Hydrogen peroxide (H_2_O_2_)—top 20 significantly correlating genes.** A total of 2477 genes were found to correlate significantly (*p* < 0.05) to H_2_O_2_-induced toxicity. Gene names, *p*-values, and Spearman r of the top 20 genes (based on *p*-value) showing positive or negative correlation with H_2_O_2_ sensitivity are displayed in this table.

H_2_O_2_ Positive Correlation			H_2_O_2_ Negative Correlation		
Gene	*p*-Value	r	Gene	*p*-Value	r
*RAB26*	4.65E-06	0.69	*SEPTIN6*	4.80E-07	−0.74
*PERP*	1.20E-05	0.67	*NREP*	6.20E-07	−0.73
*ME1*	1.39E-05	0.66	*SIRPG*	1.59E-06	−0.71
*MOCOS*	2.01E-05	0.65	*RFTN1*	4.60E-06	−0.69
*BLVRB*	2.96E-05	0.64	*CPXM1*	8.45E-06	−0.68
*ANXA9*	3.32E-05	0.64	*C4orf46*	9.29E-06	−0.67
*LCN2*	4.13E-05	0.64	*STMN1*	9.29E-06	−0.67
*CFAP300*	4.62E-05	0.63	*DTL*	1.07E-05	−0.67
*TPD52L1*	5.99E-05	0.62	*TUBB*	1.26E-05	−0.67
*TOM1L1*	7.95E-05	0.62	*CCDC28B*	1.31E-05	−0.66
*AREG*	8.91E-05	0.61	*ZNF692*	1.97E-05	−0.65
*CFB*	1.03E-04	0.61	*PET100*	3.19E-05	−0.64
*TSPAN1*	1.04E-04	0.61	*BLM*	5.16E-05	−0.63
*KLF4*	1.19E-04	0.60	*CDC25A*	5.59E-05	−0.63
*POLD4*	1.68E-04	0.59	*PLEKHO1*	6.11E-05	−0.62
*YIF1B*	1.68E-04	0.59	*PTGR3*	6.55E-05	−0.62
*SLC35A3*	1.93E-04	0.59	*SLA*	8.66E-05	−0.61
*MTFR1*	1.97E-04	0.59	*ZKSCAN4*	9.00E-05	−0.61
*DSP*	1.99E-04	0.59	*MCM10*	9.25E-05	−0.61
*OSGIN1*	1.99E-04	0.59	*H2BW2*	9.61E-05	−0.61

**Table 3:** **Hypochlorous acid (HOCl)—top 20 significantly correlating genes.** A total of 997 genes were found to correlate significantly (*p* < 0.05) to HOCl-induced toxicity. Gene names, *p*-values, and Spearman r of the top 20 genes (based on *p*-value) showing positive or negative correlation with HOCl sensitivity are displayed in this table.

HOCl Positive Correlation			HOCl Negative Correlation		
Gene	*p*-Value	r	Gene	*p*-Value	r
*ACSM3*	3.43E-03	0.48	*ZNF777*	7.22E-05	−0.62
*SLC17A9*	4.15E-03	0.47	*FZD3*	1.16E-04	−0.61
*BCL2L10*	6.15E-03	0.45	*ZNF23*	1.18E-04	−0.61
*VASP*	6.15E-03	0.45	*ZNF14*	2.11E-04	−0.59
*RPH3AL*	9.99E-03	0.43	*PEX19*	3.82E-04	−0.57
*ARHGAP18*	1.13E-02	0.42	*ZNF77*	3.20E-04	−0.57
*LIAS*	1.17E-02	0.42	*ZNF606*	3.84E-04	−0.57
*TNIK*	1.20E-02	0.42	*ZNF391*	4.00E-04	−0.57
*CD86*	1.24E-02	0.42	*ZNF728*	4.23E-04	−0.56
*PTH2*	1.43E-02	0.42	*MLLT10*	4.33E-04	−0.56
*SFXN1*	1.34E-02	0.41	*KDM2B*	5.20E-04	−0.56
*EBI3*	1.43E-02	0.41	*TP53BP2*	5.85E-04	−0.55
*CCL2*	1.49E-02	0.41	*ZNF160*	6.37E-04	−0.55
*PRAG1*	1.49E-02	0.41	*CLUAP1*	6.77E-04	−0.55
*HSPA4L*	1.68E-02	0.40	*CFDP1*	7.25E-04	−0.54
*KLK1*	1.69E-02	0.40	*FAM220A*	7.48E-04	−0.54
*ZG16B*	1.95E-02	0.39	*ZNF256*	8.75E-04	−0.54
*MYRF*	2.06E-02	0.39	*TULP4*	8.88E-04	−0.54
*GPAT3*	2.16E-02	0.39	*ZNF175*	9.85E-04	−0.53
*GPR20*	2.32E-02	0.38	*BOLA1*	1.02E-03	−0.53

## Data Availability

The underlying data of this manuscript are available from the corresponding author upon reasonable request.
